# Clinical characteristics of colonization of the amniotic cavity in women with preterm prelabor rupture of membranes, a retrospective study

**DOI:** 10.1038/s41598-022-09042-x

**Published:** 2022-03-24

**Authors:** Marian Kacerovsky, Jaroslav Stranik, Jana Matulova, Martina Chalupska, Jan Mls, Tomáš Faist, Helena Hornychova, Rudolf Kukla, Radka Bolehovska, Pavel Bostik, Bo Jacobsson, Ivana Musilova

**Affiliations:** 1 Department of Obstetrics and Gynecology, Faculty of Medicine in Hradec Kralove, University Hospital Hradec Kralove, Charles University, Sokolska 581, 500 05 Hradec Kralove, Czech Republic; 2 Biomedical Research Center, University Hospital Hradec Kralove, Hradec Kralove, Czech Republic; 3 Department of Non-Medical Studies, Faculty of Medicine in Hradec Kralove, Charles University, Hradec Kralove, Czech Republic; 4 Faculty of Medicine in Hradec Kralove, Fingerland’s Institute of Pathology, University Hospital Hradec Kralove, Charles University, Hradec Kralove, Czech Republic; 5 Faculty of Medicine in Hradec Kralove, Institute of Clinical Biochemistry and Diagnostics, University Hospital Hradec Kralove, Charles University, Hradec Kralove, Czech Republic; 6 Faculty of Medicine in Hradec Kralove, Institute of Clinical Microbiology, University Hospital Hradec Kralove, Charles University, Hradec Kralove, Czech Republic; 7 Department of Obstetrics and Gynecology, Institute of Clinical Science, Sahlgrenska Academy, University of Gothenburg, Gothenburg, Sweden; 8 Department of Obstetrics and Gynecology, Region Västra Götaland, Sahlgrenska University Hospital, Gothenburg, Sweden; 9 Department of Genetics and Bioinformatics, Domain of Health Data and Digitalization, Institute of Public Health, Oslo, Norway

**Keywords:** Diseases, Medical research, Inflammation

## Abstract

To determine the main clinical characteristics of preterm prelabor rupture of membranes (PPROM) complicated by colonization of the amniotic cavity (microbial invasion of the amniotic cavity without intra-amniotic inflammation). A total of 302 women with PPROM were included. Transabdominal amniocentesis was performed and amniotic fluid was assessed. Based of microbial invasion of the amniotic cavity and intra-amniotic inflammation (interleukin-6 ≥ 3000 pg/mL), the women were divided into following groups: intra-amniotic infection, sterile intra-amniotic inflammation, colonization of the amniotic cavity, and negative amniotic fluid. Colonization was found in 11% (32/302) of the women. The most common bacteria identified in the amniotic fluid were *Ureaplasma* spp. with a lower burden than those with intra-amniotic infection (*p* = 0.03). The intensity of intra-amniotic inflammatory response measured by interleukin-6 was higher in women with colonization than in those with negative amniotic fluid (medians: 961 pg/mL vs. 616 pg/mL; *p* = 0.04). Women with colonization had higher rates of acute inflammatory placental lesions than those with negative amniotic fluid. In PPROM, colonization, caused mainly by microorganisms from the lower genital tract, might represent an early stage of microbial invasion of the amniotic cavity with a weak intra-amniotic inflammatory response.

## Introduction

Preterm prelabor rupture of the membranes (PPROM) is defined as a rupture of the fetal membranes, with leakage of amniotic fluid before the onset of regular uterine activity earlier than 37 weeks of gestation^1,2^. PPROM is characterized by a breach in the barrier between the intra-amniotic and vaginal/cervical environments^1–4^. Therefore, PPROM can become complicated by the ascension of microorganisms from the vagina/cervix, thus leading to microorganisms in the amniotic cavity, termed microbial invasion of the amniotic cavity^5–9^. Microorganisms in the amniotic fluid are recognized by specialized pattern recognition receptors localized on the amniotic epithelium or in the amniotic fluid^10–13^. The activation of these receptors triggers a well-orchestrated intra-amniotic inflammatory response leading to the elevation of the concentrations of various inflammatory mediators (intra-amniotic inflammation), along with the attraction of immune cells into the amniotic fluid, with the ultimate goal of solving the microbial threat^14–16^.

Aside from the "classical" scenario, called intra-amniotic infection, in which the presence of microorganisms in amniotic fluid is associated with intra-amniotic inflammation, an alternative situation, where the presence of microorganisms in the amniotic fluid is not related to intra-amniotic inflammation, has been previously reported among women with PPROM^16^, with preterm labor with intact membranes^17^, and those with a short cervix^18^. This condition is typically considered as either: (1) contamination of the amniotic fluid with skin bacteria during amniotic fluid sampling and/or during pre-analytical processing of amniotic fluid samples or (2) colonization of the amniotic cavity (colonization) with microorganisms from the lower genital tract in the absence or presence of a weak intra-amniotic inflammatory response^7,19,20^.

The cause of microbial invasion of the amniotic cavity without intra-amniotic inflammation in PPROM is still under debate; however, colonization is prioritized because (1) the breach in the barrier between the lower genital tract and the amniotic cavity might enhance the ascension of vaginal/cervical bacteria into the amniotic fluid^9,21^ and (2) microbial invasion of the amniotic cavity seems to be a consequence of PPROM^22^.

In contrast with intra-amniotic infection and sterile intra-amniotic inflammation in PPROM pregnancies^16,21^, colonization is yet to be fully characterized. Therefore, it is essential to fill this knowledge gap and thoroughly investigate the clinical characteristics and significance of colonization to help clinicians deal with PPROM complications.

Therefore, the main aims of this study were (1) to characterize the microbial composition of amniotic fluid from PPROM with colonization, (2) to evaluate the main clinical characteristics of colonization, and (3) to describe the short-term morbidity of newborns from women with colonization.

## Results

Overall, 302 women with singleton pregnancies with PPROM were included in this study. The clinical definitions are shown in Table 1. Microbial invasion of the amniotic cavity and intra-amniotic inflammation were found in 23% (69/302) and 19% (58/302) of the women, respectively. Colonization, intra-amniotic infection, and sterile intra-amniotic inflammation were observed in 11% (32/302), 12% (37/302), and 7% (21/302) of the women, respectively. The remaining 70% (212/302) of the women had negative amniotic fluid. The demographic and clinical data are summarized in Table 2.

**Table 1:** The clinical definitions.

Microbial invasion of the amniotic cavity	Positive PCR analysis in amniotic fluid for *Ureaplasma* spp.*, M. hominis, C. trachomatis*, or their combination; positivity for the 16S rRNA gene in amniotic fluid; positivity for aerobic/anaerobic cultivation of the amniotic fluid; or a combination of these parameters
Intra-amniotic inflammation	Concentration of interleukin-6 in amniotic fluid, assessed using the automated electrochemiluminiscence immunoassay method, ≥ 3000 pg/mL^46^
Colonization of the amniotic fluid	Amniotic fluid with the presence microbial invasion of the amniotic cavity without intra-amniotic inflammation
Intra-amniotic infection	Amniotic fluid with the concurrent presence of microbial invasion of the amniotic cavity and intra-amniotic inflammation
Sterile intra-amniotic inflammation	Amniotic fluid with the presence of intra-amniotic inflammation without microbial invasion of the amniotic cavity
Negative amniotic fluid	Amniotic fluid without microbial invasion of the amniotic cavity and intra-amniotic inflammation
Intra-amniotic inflammatory response	Determined by the concentration of interleukin-6 in amniotic fluid
Maternal inflammatory response	Determined by the concentrations of C-reactive protein and white blood cells counts in maternal blood
Acute histological chorioamnionitis	Histological grades 3–4 for the chorion-decidua, and/or grades 3–4 for the chorionic plate, and/or grades 1–4 for the umbilical cord, and/or grades 1–4 for the amnion^44^
Funisitis	Histological grades 1–4 for the umbilical cord^44^
Acute inflammation of the amnion	Histological grades 1–4 for the amnion^44^
Compound neonatal morbidity	the need for intubation, and/or respiratory distress syndrome, and/or transient tachypnea of newborns, and/or bronchopulmonary dysplasia, and/or retinopathy of prematurity, and/or intraventricular hemorrhage, and/or necrotizing enterocolitis, and/or intestinal perforation, and/or early-onset sepsis, and/or late-onset sepsis, and/or retinopathy from prematurity, and/or neonatal death before hospital discharge^24^

**Table 2:** Demographical and clinical characteristics of women with preterm prelabor rupture of membranes with respect to the presence of intra-amniotic infection, sterile intra-amniotic inflammation, colonization of the amniotic cavity and negative amniotic fluid.

	Intra-amniotic infection (n = 37)	Sterile intra-amniotic inflammation (n = 21)	Colonization of the amniotic cavity (n = 32)	Negative amniotic fluid (n = 212)	*p *value	*p *value^1^	*p *value^2^	*p *value^3^
Maternal age [years, median (IQR)]	31 (26–37)	33 (28–37)	31 (24–37)	31 (27–35)	0.84	0.60	0.50	0.53
Primiparous [number (%)]	15 (52%)	6 (41%)	13 (41%)	130 (61%)	**0.002**	0.41	0.99	**0.03**
Pre-pregnancy body mass index [kg/m^2^, median (IQR)]	23.6 (21.0–26.9)	25.1 (19.9–28.3)	24.0 (19.7–27.1)	24.6 (21.8–28.1)	0.42	0.76	0.87	0.53
Gestational age at sampling [weeks + days, median (IQR)]	29 + 3 (25 + 4–33 + 0)	30 + 1 (24 + 4–33 + 4)	33 + 6 (31 + 6–35 + 3)	34 + 2 (32 + 0–35 + 2)	** < 0.0001**	** < 0.0001**	**0.0006**	0.70
Gestational age at delivery [weeks + days, median (IQR)]	31 + 1 (27 + 1–33 + 2)	32 + 2 (29 + 2–34 + 0)	34 + 2 (32 + 6–35 + 4)	34 + 4 (33 + 0–35 + 5)	** < 0.0001**	** < 0.0001**	**0.0009**	0.84
Latency from PPROM to amniocentesis [hours, median (IQR)]	4 (2–12)	5 (3–15)	4 (2–8)	4 (2–7)	0.42	0.78	0.32	0.70
Latency from amniocentesis to delivery [hours, median (IQR)]	64 (25–168)	125 (19–424)	65 (28–126)	51 (14–159)	0.26	0.86	0.16	0.74
Amniotic fluid IL-6 concentrations [pg/mL, median (IQR)]	20,599 (9605–42,876)	6664 (3342–11,039)	961 (510–1566)	616 (322–1155)	** < 0.0001**	** < 0.0001**	** < 0.0001**	**0.04**
CRP levels at admission [mg/L, median (IQR)]	9.3 (3.8–27.3)	6.5 (2.8–15.3)	4.3 (2.4–8.3)	5.2 (2.9–9.2)	**0.004**	**0.003**	0.14	0.38
WBC count at admission [× 10^9^ L, median (IQR)]	14.6 (10.9–17.8)	11.4 (9.9–15.2)	11.3 (9.6–13.9)	12.2 (10.2–14.9)	**0.04**	**0.006**	0.41	0.11
Administration of corticosteroids [number (%)]	32 (87%)	18 (86%)	22 (69%)	145 (68%)	0.06	0.99	0.09	**0.03**
Administration of antibiotics [number (%)]	37 (100%)	20 (95%)	31 (97%)	212 (100%)	**0.02**	0.36	0.47	–
Spontaneous vaginal delivery [number (%)]	24 (65%)	11 (52%)	22 (69%)	148 (70%)	0.42	0.41	0.80	0.57
Forceps delivery [number (%)]	0 (0%)	0 (0%)	0 (0%)	2 (1%)	0.84	–	–	1.00
Cesarean section [number (%)]	13 (35%)	10 (48%)	10 (31%)	62 (29%)	0.34	0.41	0.80	0.56
Birth weight [grams, median (IQR)]	1590 (1020–2220)	1770 (1195–2080)	2225 (1783–2535)	2290 (1920–2618)	** < 0.0001**	**0.0001**	**0.002**	0.74
Apgar score < 7; 5 min [number (%)]	6 (16%)	2 (10%)	1 (3%)	4 (2%)	** < 0.0001**	0.70	0.11	**0.0002**
Apgar score < 7; 10 min [number (%)]	4 (11%)	0 (0%)	1 (3%)	3 (1%)	**0.01**	0.29	0.36	**0.01**

Abbreviations:

CRP: C-reactive protein, IL: interleukin, WBC: White blood cells.

Continuous variables were compared using a nonparametric Kruskal–Wallis or Mann–Whitney *U* test. Categorical variables were compared using chi-square test or Fisher’s exact test. Statistically significant results are marked in bold. Continuous variables are presented as median (interquartile range) and categorical as number (%).

*p* value—comparison among women with intra-amniotic infection, with sterile intra-amniotic infection, with colonization of the amniotic cavity, and with negative amniotic fluid.

*p* value^1^—comparison between women with colonization of the amniotic cavity and with intra-amniotic infection.

*p* value^2^—comparison between women with colonization of the amniotic cavity and with sterile intra-amniotic inflammation.

*p* value^3^—comparison between women with colonization of the amniotic cavity and with negative amniotic fluid.

### Amniotic fluid microorganisms

**Figure 1:**
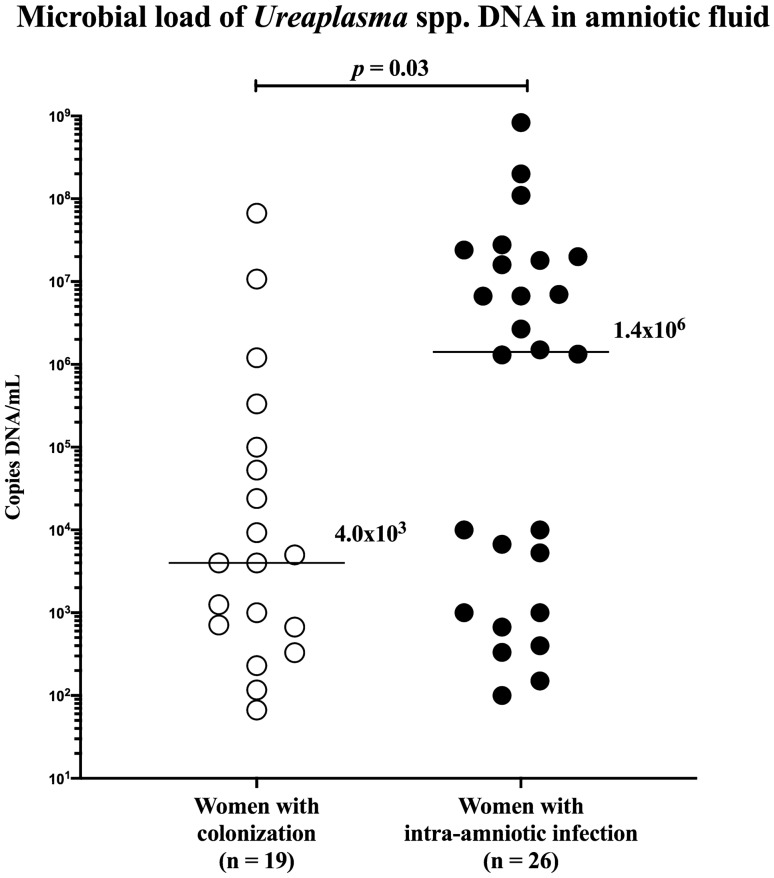
Comparison of the load of *Ureaplasma* spp. DNA in the amniotic fluid between women with colonization and those with intra-amniotic infection. The median values are marked.

All microorganisms identified in the amniotic fluid from women with colonization and intra-amniotic infection are listed in Table 3. Polymicrobial findings were revealed in 32% (10/32) of the women with colonization. There was no difference in the rate of polymicrobial findings in the amniotic fluid between the women with colonization and those with intra-amniotic infection (intra-amniotic infection, 19% [7/37]; *p* = 0.27). The most common microorganism identified in the amniotic fluid from women with colonization and intra-amniotic infection was *Ureaplasma* spp. (colonization: 59% [19/32], intra-amniotic infection: 70% [26/37]; *p* = 0.45). Women with colonization had a lower load of *Ureaplasma* spp. DNA in the amniotic fluid than women with intra-amniotic infection (colonization: median 4 × 10^3^, IQR 6.7 × 10^2^ – 1.0 × 10^5^ vs. infection: median 1.4 × 10^6^, IQR 1.0 × 10^3^ – 1.9 × 10^7^; *p* = 0.03; Fig. 1).

**Table 3:** The microbial species identified in the amniotic fluid of PPROM pregnancies with colonization of the amniotic cavity and intra-amniotic infection.

Colonization of the amniotic cavity (n = 32)	Intra-amniotic infection (n = 37)
*Ureaplasma* spp. + *Gardnerella vaginalis* + *Prevotella disiens* [1 (3%]	*Ureaplasma* spp. + *Gardnerella vaginalis* + *Aerococcus christensenii* [1 (3%)]
*Ureaplasma* spp. + *Lactobacillus iners* + *Sneathia sanguinegens* [1 (3%]	*Ureaplasma* spp. + *Dialister micraerophilus* + *Atopobium vaginae* [1 (3%)]
*Ureaplasma* spp. + *Mycoplasma hominis* [2 (7%]	*Ureaplasma* spp. + *Chlamydia trachomatis* + *Fusobacterium nucleatum* [1 (3%)]
*Ureaplasma* spp. + *Gardnerella vaginalis* [1 (3%]	*Ureaplasma* spp. + *Escherichia coli* [1 (3%)]
*Ureaplasma* spp. + *Streptococcus mitis* [1 (3%)]	*Ureaplasma* spp. + *Chlamydia trachomatis* [1 (3%)]
*Ureaplasma* spp. + *Streptococcus oralis* [1 (3%)]	*Ureaplasma* spp. + *Streptococcus anginosus* [1 (3%)]
*Ureaplasma spp.* [12 (38%)]	*Ureaplasma* spp. [20 (54%)]
*Lactobacillus gasseri* + *Gardnerella vaginalis* + *Corynebacterium* spp. + *Prevotella bivia* [1 (3%)]	*Streptococcus oralis* + *Streptococcus anginosus* + *Campylobacter ureolyticus* [1 (3%)]
*Staphylococcus epidermidis* + *Dermabacter hominis* + *Corynebacterium tuberculostearicum* [1 (3%)]	*Haemophilus influenzae* [4 (11%)]
*Gardnerella vaginalis* + *Sneathia sanguinegens* [1 (3%)]	*Anaerococcus tetradius* [1 (3%)]
*Chlamydia trachomatis* [1 (3%)]	*Lactobacillus jensenii* [1 (3%)]
*Clostridium perfringens* [1 (3%)]	*Gardnerella vaginalis* [1 (3%)]
*Escherichia coli* [1 (3%)]	*Enterococcus faecalis* [1 (3%)]
*Mycoplasma hominis* [1 (3%)]	*Peptoniphilus indolicus* [1 (3%)]
*Moraxella osloensis* [1 (3%)]	*Streptococcus anginosus* [1 (3%)]
*Peptostreptococcus stomatis* [1 (3%)]	
*Sneathia sanguinegens* [1 (3%)]	
*Streptococcus agalactiae* [1 (3%)]	
*Streptococcus mitis* [1 (3%)]	
*Streptococcus sanguinis* [1 (3%)]	

Categorical data are presented as number (%).

### Gestational age at rupture of membranes and latency interval between rupture of membranes and delivery

**Figure 2:**
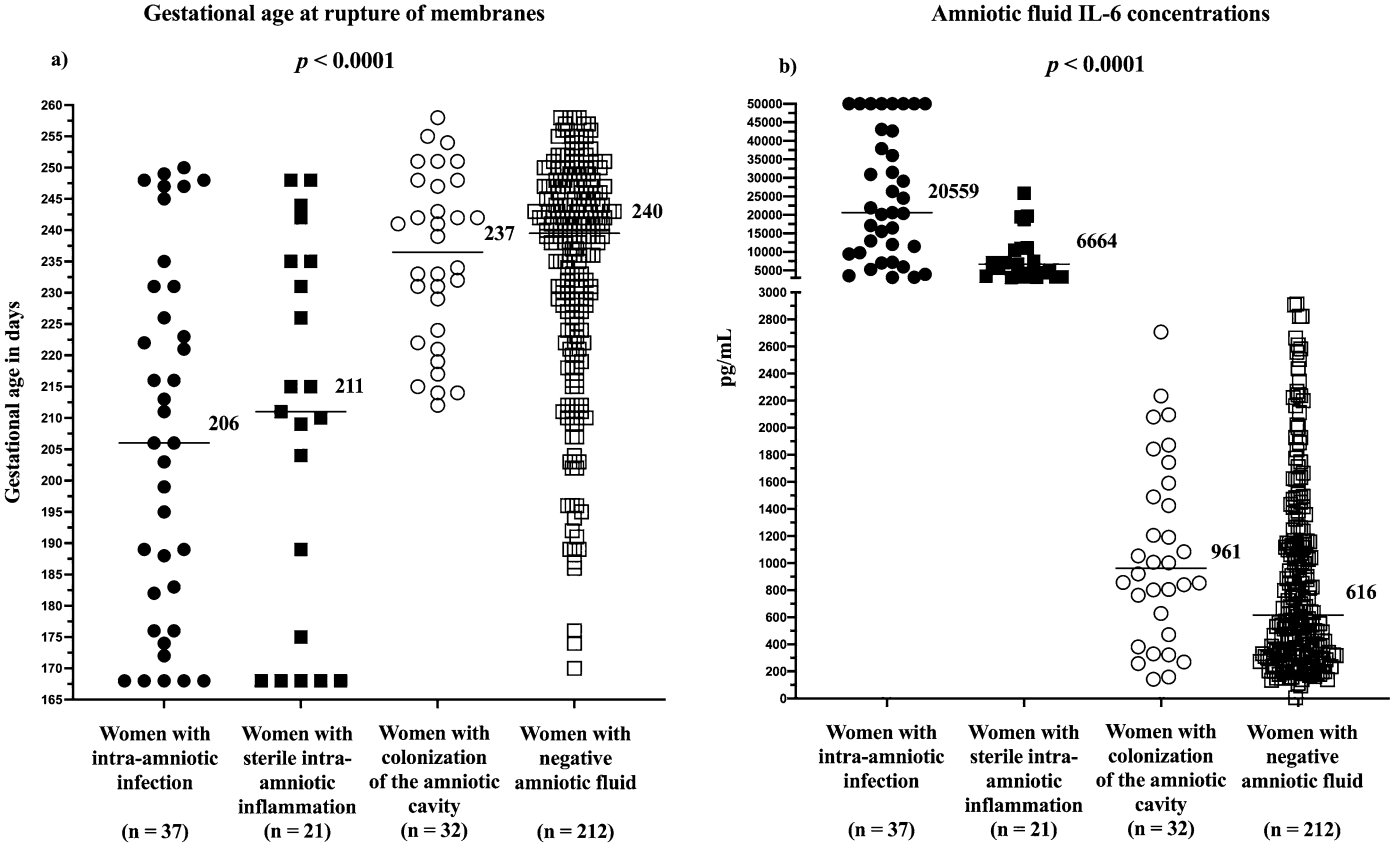
Gestational ages at rupture of membranes (**a**) and concentrations of amniotic fluid interleukin-6 (**b**) in women with preterm prelabor rupture of membranes with respect to intra-amniotic infection, sterile intra-amniotic inflammation, colonization of the amniotic cavity, and negative amniotic fluid. The median values are marked.

Gestational age at rupture of membranes differed among the subgroups of the women (*p* < 0.0001; Fig. 2a). Women with colonization had a higher gestational age at rupture of membranes (median 33 + 6, IQR 31 + 6 – 35 + 3) than women with intra-amniotic infection (median 29 + 3, IQR 26 + 0 – 31 + 3; *p* < 0.0001) and those with sterile intra-amniotic inflammation (median 30 + 1, IQR 24 + 4 – 30 + 5; *p* = 0.0006; Fig. 2a), but similar to those with negative amniotic fluid (median 34 + 2, IQR 32 + 0 – 35 + 2; *p* = 0.97).

**Figure 3:**
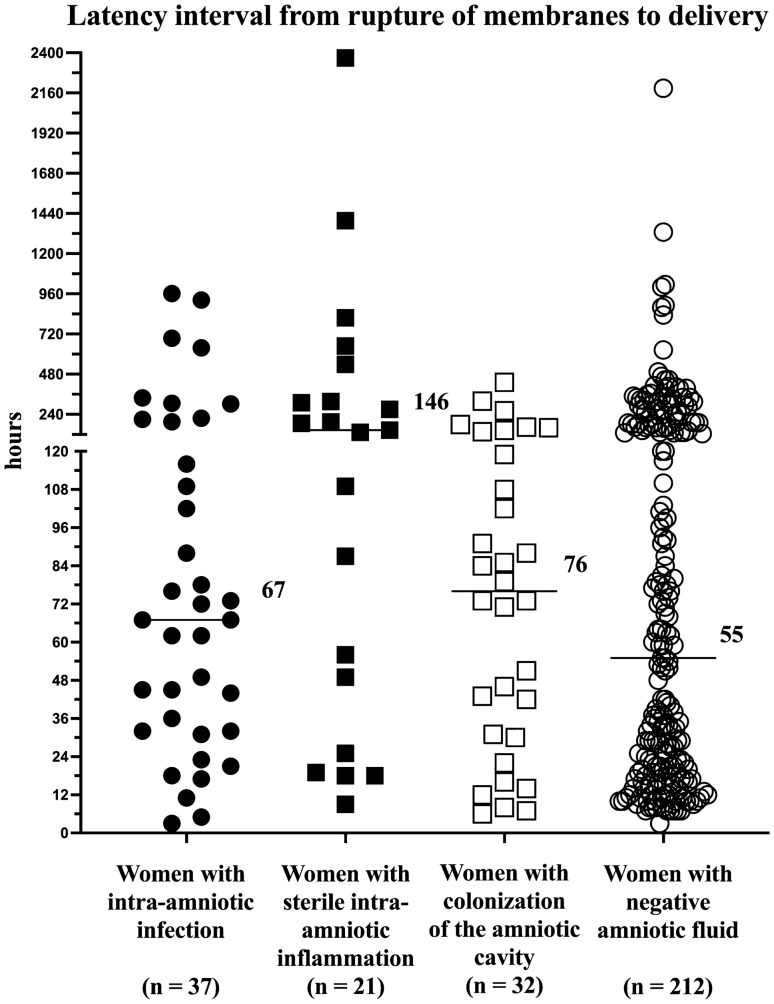
Latency interval (hours) from rupture of membranes to delivery in women with preterm prelabor rupture of membranes with respect to intra-amniotic infection, sterile intra-amniotic inflammation, colonization of the amniotic cavity, and negative amniotic fluid. The median values are marked.

Latency interval (hours) from rupture of membranes to delivery in women with preterm prelabor rupture of membranes with respect to intra-amniotic infection, sterile intra-amniotic inflammation, colonization of the amniotic cavity, and negative amniotic fluid is shown in Fig. 3. No difference in the latency interval from rupture of membranes to delivery was found between colonization and negative amniotic fluid (colonization: median 76 h, IQR 30–132 vs. negative amniotic fluid: median 55 h, IQR 19–179; *p* = 0.76). However, women with colonization had a shorter latency interval than women with sterile intra-amniotic inflammation (median 146 h, IQR 37–428; *p* = 0.03). Comparisons with women with intra-amniotic infection were not made due to a different management approach (active management beyond the 28th week of gestation).

### Intensity of intra-amniotic and maternal inflammatory responses

Concentrations of interleukin (IL)-6 in amniotic fluid differed among the subgroups of the women (*p* < 0.0001; Fig. 2b). Women with colonization had a higher concentration of IL-6 in the amniotic fluid (median: 961 pg/mL, IQR 510–1566) than those with negative amniotic fluid (median 616 pg/mL, IQR 322–2914; *p* = 0.04; Fig. 2b).

Women with colonization had a lower intensity of maternal inflammatory response measured by C-reactive protein (CRP) and white blood cell (WBC) count than women with intra-amniotic infection (CRP—colonization: median 4.3 mg/L, IQR 2.4–8.4 vs. infection: median 13.8 mg/L, IQR 3.8–27.4; *p* = 0.003; WBC—colonization: median 11.3 × 10^9^ L, IQR 9.6–13.9 vs. infection: median 14.6 × 10^9^ L, IQR 10.9–17.8; *p* = 0.006). No differences in maternal inflammatory response were found in the comparisons between women with sterile intra-amniotic inflammation (CRP, median 4.3 mg/L; IQR 2.4–8.4; *p* = 0.14; WBC: median 11.4 × 10^9^ L; *p* = 0.41) and negative amniotic fluid (CRP: median 5.2 mg/L, IQR 3.0–9.2; *p* = 0.38; WBC: median 12.2 × 10^9^ L, IQR 10.2–14.9; *p* = 0.11).

### Acute inflammatory lesions in the placenta

**Figure 4:**
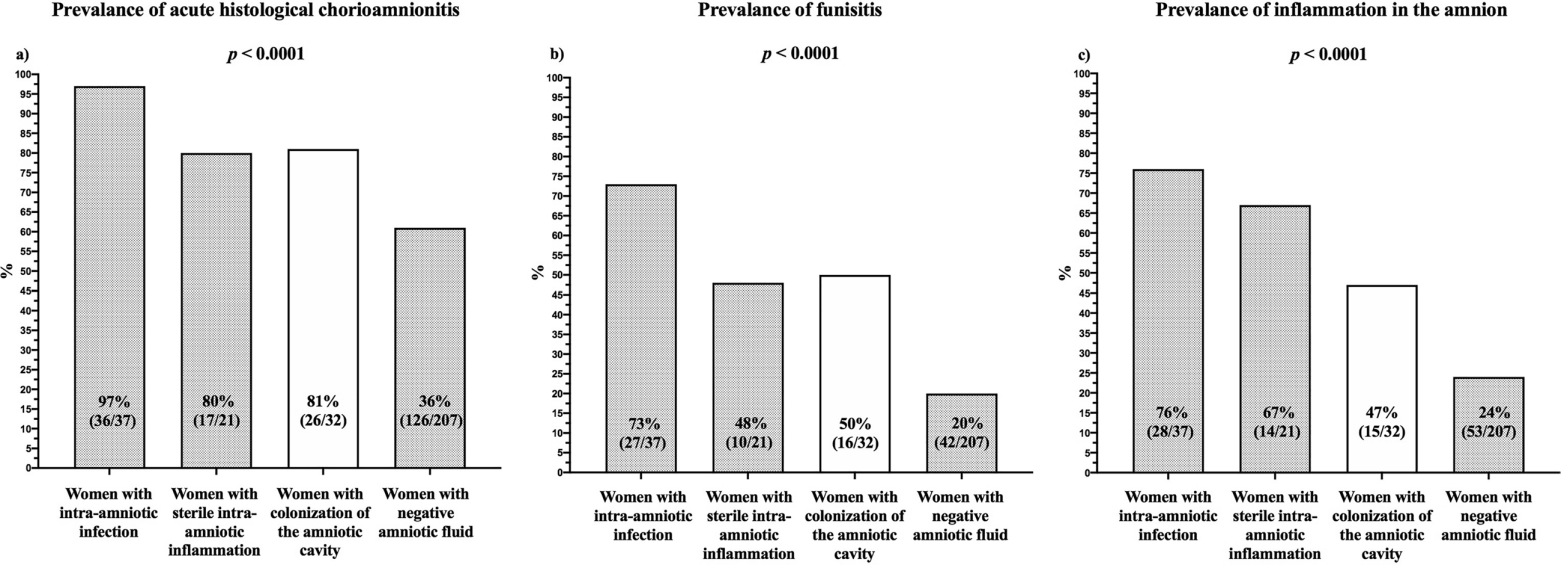
Prevalence of acute histological chorioamnionitis (**a**), funisitis (**b**), and inflammation of the amnion (**c**) in PPROM pregnancies based on intra-amniotic infection, sterile intra-amniotic inflammation, colonization of the amniotic cavity, and negative amniotic fluid.

The results regarding acute inflammatory lesions in the placenta were available for 98% (297/302) of the women (five missing findings were from women with negative amniotic fluid). The rates of acute histological chorioamnionitis (HCA), funisitis and inflammation of the amnion differed among the subgroups of the women (*p* < 0.0001 for all, Table 4). Women with colonization had lower rates of HCA, funisitis, and inflammation of the amnion (HCA: 81% [26/32]; funisitis: 50% [16/32]; and amnion: 47% [15/32]; Fig. 4) than women with intra-amniotic infection (HCA: 97% [36/37], *p* = 0.04; funisitis: 73% [27/37], *p* = 0.08; amnion: 76% [28/37], *p* = 0.02; Fig. 4); however, the difference in funisitis reached just borderline statistical significance. Women with colonization had higher rates of HCA, funisitis, and inflammation of the amnion than those with negative amniotic fluid (HCA: 61% [126/207], *p* = 0.03; funisitis: 20% [42/207], *p* = 0.0007; and amnion: 24% [53/207], *p* = 0.007; Fig. 4). No differences were found between women with colonization and sterile intra-amniotic inflammation (HCA: 80% [17/21], *p* = 1.00; funisitis: 48% [10/21], *p* = 1.00; amnion: 67% [14/21], *p* = 0.17; Fig. 4).

**Table 4:** Short-term neonatal morbidity of newborns from pregnancies with PPROM with respect to intra-amniotic infection, sterile intra-amniotic inflammation, colonization, and negative amniotic fluid.

Characteristic	Intra-amniotic Infection (n = 37)	Sterile intra-amniotic inflammation (n = 21)	Colonization of the amniotic cavity (n = 32)	Negative amniotic fluid (n = 212)	*p *value^1^	*p *value^1#^	*p *value^2^	*p *value^2#^	*p *value^3^	*p *value^3#^
Need for intubation [number (%)]	2 (5%)	0 (0%)	1 (3%)	5 (2%)	1.00	0.99	1.00	0.34	0.57	0.73
Respiratory disorders	19 (51%)	10 (48%)	7 (22%)	56 (26%)	**0.01**	0.94	0.07	0.89	0.67	0.59
Respiratory distress syndrome [number (%)]	19 (51%)	9 (43%)	6 (19%)	43 (20%)	**0.006**	0.72	0.07	0.96	1.00	0.91
Transient tachypnea of newborns	0 (0%)	1 (5%)	1 (3%)	13 (6%)	0.46	0.15	1.00	0.70	0.70	0.51
Intraventricular hemorrhage [number (%)]	7 (19%)	5 (24%)	3 (9%)	18 (9%)	0.32	0.89	0.24	0.83	0.74	0.83
intraventricular hemorrhage grade I-II [number (%)]	5 (14%)	5 (24%)	3 (9%)	17 (8%)	0.72	0.81	0.24	0.83	0.74	0.77
Intraventricular hemorrhage grade III-IV [number (%)]	2 (5%)	0 (0%)	0 (0%)	1 (1%)	0.50	0.45	–	–	1.00	0.72
Necrotizing enterocolitis [number (%)]	1 (3%)	1 (5%)	0 (0%)	0 (0%)	1.00	0.98	0.40	0.89	–	–
Intestinal perforation [number (%)]	1 (3%)	1 (5%)	0 (0%)	0 (0%)	1.00	0.89	0.40	0.89	–	–
Early-onset sepsis [number (%)]	3 (8%)	2 (10%)	1 (3%)	3 (2%)	0.62	0.85	0.55	0.40	0.43	0.47
Late-onset sepsis [number (%)]	1 (3%)	0 (0%)	0 (0%)	0 (0%)	1.00	0.98	–	–	–	–
Pneumonia [number (%)]	2 (5%)	0 (0%)	1 (3%)	1 (1%)	1.00	0.69	1.00	0.34	0.25	0.11
Bronchopulmonary dysplasia [number (%)]	9 (24%)	3 (14%)	0 (0%)	5 (2%)	**0.003**	0.78	0.06	0.83	1.00	0.38
Retinopathy of prematurity [number (%)]	4 (11%)	2 (10%)	0 (0%)	4 (2%)	0.12	0.97	0.15	0.88	1.00	0.45
Death before discharge [number (%)]	3 (8%)	0 (0%)	0 (0%)	2 (1%)	0.24	0.84	–	–	1.00	0.60
Compound neonatal morbidity [number (%)]	22 (60%)	12 (57%)	10 (31%)	65 (31%)	**0.03**	0.58	0.09	0.95	1.00	0.82

Variables were compared using the Fisher’s exact test and are presented as number (%). Statistically significant results are marked in bold.

*p-*value^1^—comparison between women with colonization of the amniotic cavity and with intra-amniotic infection.

*p-*value^2^—comparison between women with colonization of the amniotic cavity and with sterile intra-amniotic inflammation.

*p-*value^3^—comparison between women with colonization of the amniotic cavity and with negative amniotic fluid.

^#^ the results were adjusted for gestational age at sampling.

### Short-term neonatal outcomes

The short-term outcomes of newborns from PPROM pregnancies are presented in Table 4. No differences in the rates of short-term neonatal outcomes were found between colonization and negative amniotic fluid. Colonization was related to a lower compound neonatal morbidity than intra-amniotic infection as per crude analysis, but this was not so after adjusting for gestational age at delivery.

## Discussion

The principal findings of this study are as follows: (1) colonization was found in 11% of PPROM pregnancies; (2) *Ureaplasma* spp. were the most common amniotic fluid microorganisms associated with colonization; however, the burden was lower than that found in women with intra-amniotic infection; (3) the gestational age at rupture of membranes in women with colonization was similar to that of women with negative amniotic fluid but higher than in those with intra-amniotic inflammation; (4) the intensity of intra-amniotic inflammatory response (IL-6 concentrations) was higher in women with colonization than in those with negative amniotic fluid; (5) the rates of acute inflammatory placental lesions was higher in women with colonization than in those with negative amniotic fluid; and (6) short-term neonatal morbidity did not differ between newborns from PPROM with colonization and those with negative amniotic fluid.

Microbial invasion of the amniotic cavity represents a condition with two clinical phenotypes: (1) intra-amniotic infection when intra-amniotic inflammation is present and (2) colonization/contamination when intra-amniotic inflammation is absent^16,19,20^. The rates of these phenotypes might differ among cohorts of women with PPROM due to differences in distribution of gestational age at rupture of membranes, race/ethnicity, and latency interval between ruptured membranes and sampling. In this study, the rates of intra-amniotic infection and colonization were almost equal (12% and 11%, respectively). The rate of colonization found in this study was similar to that reported previously (12%) in the study by Romero et al.^16^; however, the rate of intra-amniotic infection was lower than that reported previously (12% vs. 29%)^16^. These results show that colonization can be responsible for a significant amount (30–50%) of cases with microbial invasion of the amniotic cavity in PPROM pregnancies. This observation is of clinical significance. However, we must be aware that cases with colonization remain clinically hidden unless the assessment of both microbial invasion of the amniotic cavity and intra-amniotic inflammation is employed. This is in direct contrast to cases with intra-amniotic infection (certainly, along with those with sterile intra-amniotic inflammation), for which a less expensive and less time-consuming assessment of intra-amniotic inflammation is sufficient.

There is solid evidence that *Ureaplasma* spp. are the most common pathogens in the amniotic fluid from PPROM^16,23^. These low virulent commensal bacteria, commonly found in the vagina and/or cervix are among the smallest self-replicating microorganisms that can grow independently^24–26^. In this study, the rate of *Ureaplasma* spp. in women with colonization reached 60%. In addition, a vast majority of microorganisms, other than *Ureaplasma* spp., identified in the amniotic fluid from this subset of women, were bacteria commonly present in the vagina, cervix, or rectal niche. These observations support that ascension of the bacteria from the lower genital tract might cause colonization in women with PPROM.

The microbial burden of the amniotic fluid with *Ureaplasma* spp. varies from hundreds to billions of copies of DNA per milliliter of amniotic fluid in PPROM and it is higher in women with intra-amniotic infection than in those with colonization^21^. Accordingly, a lower burden of *Ureaplasma* spp. was observed in women with colonization than in those with intra-amniotic infection in this study. Given the dose-dependent relationship between intra-amniotic inflammatory response (measured by IL-6 concentrations) and the amniotic fluid burden of *Ureaplasma* spp. in PPROM pregnancies^21,27,28^, it is likely that women with colonization, caused by *Ureaplasma* spp., could not elicit an intra-amniotic inflammatory response intense enough to pass a clinical threshold for intra-amniotic inflammation. In addition, clinicians should be aware of the following facts: (1) intra-amniotic inflammation is expressed as a categorical condition (present/absent) and (2) IL-6 is a physiological constituent of amniotic fluid (measurable concentrations of IL-6 in all PPROM pregnancies. Collectively, distinguishing between the early-stage of the "classical" intra-amniotic inflammatory response to a microorganism with a weak inflammatory response and just the presence of microorganisms in the amniotic fluid with the absence of an intra-amniotic inflammatory response is at this stage almost impossible.

There is evidence that the rate of intra-amniotic infection in PPROM pregnancies decreases with advanced gestational age^16,21^. However, colonization has been previously described only in the subset of PPROM with gestational ages between 25 and 33 weeks^16^. In contrast, colonization in PPROM was found in this study only beyond the 30th week of gestation. In addition, the range of gestational ages at rupture of membranes among women with colonization was very narrow (only 46 days) compared to those with intra-amniotic infection, sterile intra-amniotic inflammation, and negative amniotic fluid, in which the ranges were almost two-fold higher. These findings might extend our previous observation, where an intensive intra-amniotic inflammatory response to amniotic fluid bacteria, characterized by concentrations of multiple inflammatory-related proteins in amniotic fluid, was not found beyond gestational 32 weeks^29^.

The intra-amniotic inflammatory response is associated with elevated concentrations of various cytokines, chemokines, and other inflammation-related proteins and lipids in the amniotic fluid^30,31^. This condition is typically followed by the attraction of neutrophils towards the amniotic cavity from the intervillous space of the placenta into the chorionic plate and/or from the decidua into fetal membranes, leading to the development of acute inflammatory lesions in the placenta^32^. In this study, both intra-amniotic inflammatory responses, measured by concentrations of IL-6 in the amniotic fluid and the presence of acute inflammatory placental lesions, were assessed. Collectively, women with colonization had a higher intensity of intra-amniotic inflammatory response and rates of acute inflammatory placental lesions than women with negative amniotic fluid. This observation further supports the hypothesis mentioned above that colonization in PPROM represents an early stage of microbial invasion of the amniotic cavity with a weak intra-amniotic inflammatory response that is not intense enough to pass a clinical threshold for intra-amniotic inflammation.

It remains debatable whether the microbial invasion of the amniotic cavity and/or intra-amniotic inflammation, after correction for gestational age at delivery, is associated with worse neonatal outcomes^16,21,33^. To extend this knowledge, the selected aspects of short-term neonatal morbidity were investigated in this study. After adjusting for gestational age at delivery, no differences were found between newborns from PPROM with colonization and negative amniotic fluid.

This study had several strengths. First, a relatively large cohort of women with singleton pregnancies complicated by a well-defined clinical phenotype of spontaneous preterm labor (PPROM) with available information about the intra-amniotic environment and histopathology of the placenta was used. Second, a thorough assessment of microbial invasion of the amniotic cavity consisting of specific PCR for *Ureaplasma* spp., *M. hominis,* and *Ch. Trachomatis*; non-specific PCR for 16S rRNA; and aerobic/anaerobic cultivation were employed in this study. Finally, short-term neonatal outcomes were available for all newborns in the PPROM cohort.

This study also has limitations that are worth mentioning. First, this study consisted of a homogeneous population of Caucasian women living in the eastern part of the Czech Republic. This prevents the findings of this study from being generalized to populations with racial/ethnic disparities. Second, only loads of *Ureaplasma* spp. DNA, but not all bacterial DNA, in the amniotic fluid were evaluated in this study. This shortcoming prevented us from assessing whether the burdens of amniotic fluid bacteria other than *Ureaplasma* spp. differ between women with colonization and those with intra-amniotic inflammation. However, in a recent study on women with preterm labor with intact membranes, microbial and fungal burdens in the amniotic fluid were lower in those with microbial invasion of the amniotic cavity without intra-amniotic inflammation than in those with intra-amniotic inflammation^17^. Last, the long-term outcomes of infants from PPROM pregnancies were not available.

In conclusion, colonization in PPROM, caused mainly by microorganisms from the lower genital tract, might represent an early stage of microbial invasion of the amniotic cavity with a weak intra-amniotic inflammatory response that is not intense enough to pass a clinical threshold for intra-amniotic inflammation.

## Methods

This retrospective study included all pregnant women admitted to the Department of Obstetrics and Gynecology of the University Hospital Hradec Kralove, Czech Republic, between December 2018 and July 2021, who met the following criteria: (1) age ≥ 18 years, (2) PPROM between gestational ages 24 + 0 and 36 + 6 weeks, (3) singleton pregnancy, and (4) transabdominal amniocentesis. The exclusion criteria were as follows: (1) pregnancy-related and other medical complications (e.g., pregestational diabetes, gestational hypertension, and preeclampsia), (2) congenital or chromosomal fetal abnormalities, (3) signs of fetal hypoxia, and (4) significant vaginal bleeding.

Gestational age was determined based on a first-trimester ultrasound scan. PPROM was diagnosed based on visual confirmation of amniotic fluid pooling in the posterior vaginal fornix using a sterile speculum for examination, or by determination of the presence of insulin-like growth factor-binding protein in the vaginal fluid (Actim PROM test; Medix Biochemica, Kauniainen, Finland) if uncertainty remained after the clinical examination.

Maternal blood and amniotic fluid samples were obtained at the time of admission before the administration of corticosteroids, antibiotics, or tocolytics. The performance of transabdominal amniocentesis to assess the intra-amniotic environment has been a part of the department's standard clinical management of women with PPROM. Women with PPROM were further managed based on amniotic fluid test results. Those with confirmed intra-amniotic inflammation received intravenous clarithromycin for seven days unless delivery occurred. Women without intra-amniotic inflammation were treated with intravenous benzylpenicillin (intravenous clindamycin in case of penicillin allergy) for seven days unless delivery occurred. Once the final results regarding microbial invasion of the amniotic cavity from cultivation and/or PCR were known, the attending clinician decided to modify the antibiotic therapy. Corticosteroids (betamethasone) were administered to those at gestational ages between 24 + 0 and 34 + 6 weeks to accelerate lung maturation. Tocolysis (atosiban) was used in those who developed regular uterine activity during the course of corticosteroid therapy or within 24 h after their administration.

Women with proven intra-amniotic infection beyond the 28th gestational week were managed actively (labor was induced, or an elective cesarean section was performed after completing corticosteroid treatment within 72 h of membrane rupture for pregnancies before 34 weeks of gestational age, and once an intra-amniotic infection was confirmed for those beyond 34 weeks). The remaining women with PPROM were managed expectantly.

After delivery, the placenta, fetal membranes, and umbilical cord were fixed in 10% neutral buffered formalin and sent for histopathological evaluation.

This study was approved by the approved by the Institutional Review Board of the University Hospital Hradec Kralove (June 2017, No. 201706 S15P). All experiments were performed in accordance with relevant guidelines and regulations. All participants in the study were Caucasian. Informed consent was obtained from all participants.

Amniotic fluid samples^19,34^, cervical fluid samples^19,35^, and clinical and demographical data^36,37^ from some women from this cohort were used in our previous studies. Therefore, microbial results from amniotic fluid were shown, in part, in these publications^19,34–37^.

### Amniotic fluid sampling

During free-hand ultrasonography-guided amniocentesis, performed at the time of admission before administration of corticosteroids, antibiotics, or tocolytics, approximately 3 mL of amniotic fluid was aspirated and used for the assessment of IL-6 concentration; PCR for *Ureaplasma* spp., *Mycoplasma hominis*, and *Chlamydia trachomatis*; 16S rRNA gene; and aerobic/anaerobic cultivation. The remaining amniotic fluid was further processed, and aliquots and pellets were frozen and used for research purposes. Details about amniotic fluid sampling were described in our previous publication^20^.

### Assessment of amniotic fluid IL-6

The concentration of IL-6 in the amniotic fluid was assessed using the automated electrochemiluminescence immunoassay method with the immuno-analyzer Cobas e602 (Roche Diagnostics, Basel, Switzerland)^38^. The measurable range was 1.5–5000 pg/mL, which could be extended to 50,000 pg/mL with a tenfold dilution of the sample. The coefficients of variation for the inter-assay and intra-assay precisions were both < 10%.

### Detection of Ureaplasma spp., M. hominis, and C. trachomatis in the amniotic fluid

A commercial AmpliSens® *C. trachomatis*/*Ureaplasma*/*M. hominis*-FRT kit (Federal State Institution of Science, Central Research Institute of Epidemiology, Moscow, Russia) was used to detect the DNA of *Ureaplasma* spp., *M. hominis*, and *Ch. trachomatis* using a single PCR tube for each fluid. The details have been described previously^19,39,40^. The level of *Ureaplasma* spp. (copies/mL) was determined using an absolute quantification technique that uses an external calibration curve. Plasmid DNA (pCR3, Invitrogen, Carlsbad, CA, USA) was used to prepare the calibration curve.

### PCR detection and sequencing of 16S rRNA gene

Bacterial DNA was identified by PCR targeting the 16S rRNA using the following primers: 5-CCAGACTCCTACGGGAGGCAG-3 (V3 region) and 5-ACATTTCACAACACGAGC-GACGA-3 (V6 region)^41^. The details have been described previously^19,39,40^. The bacteria were then typed using the sequences obtained from BLAST® and SepsiTestTM BLAST^20^.

### Aerobic and anaerobic cultures of amniotic fluid

The amniotic fluid samples were cultured on Columbia agar with sheep's blood, *Gardnerella vaginalis* selective medium, MacConkey agar, *Neisseria*-selective medium (modified Thayer–Martin medium), Sabouraud agar, and Schaedler anaerobe agar. The details have been described previously^19,39^.

### Assessment of maternal blood CRP and WBC

A maternal blood sample was obtained by venipuncture of the cubital vein and sent to the laboratory immediately following sampling to assess the CRP and WBC count. Details about maternal blood CRP and WBC count are described in our previous publications^23,42,43^.

### Histopathology of the placenta

Tissue samples were obtained from the placenta (at least two samples), fetal membranes (one sample from the free margin of the membranes, one from the central part of the membranes, and one from the membranes at the marginal part of the placenta), and umbilical cord (usually one sample), which were routinely processed and embedded in paraffin. Sections of the tissue blocks were stained with hematoxylin and eosin. The degree of neutrophil infiltration was evaluated separately in the free membranes (amnion and chorion-decidua), chorionic plate, and umbilical cord according to the criteria provided by Salafia et al.^44^. Histopathological examinations were performed by a single pathologist (HH) who was blinded to the clinical status of the women.

### Definitions of selected aspects of short-term neonatal morbidity

Maternal and perinatal medical records were reviewed by five investigators (JM, JS, MCH, TF, and MK). Data regarding short-term neonatal morbidity were reviewed for all the newborns. Definitions of selected aspects of short-term neonatal morbidity have been described in our previous publications^20,45^.

### Clinical definitions

Microbial invasion of the amniotic cavity was determined based on positive PCR analysis for *Ureaplasma* spp.*, M. hominis, C. trachomatis*, or their combination; positivity for the 16S rRNA gene; positivity for aerobic/anaerobic cultivation of the amniotic fluid; or a combination of these parameters. Intra-amniotic inflammation was defined as an amniotic fluid with an IL-6 concentration of greater than or equal to 3000 pg/mL^46^. Colonization was defined as the presence microbial invasion of the amniotic cavity without intra-amniotic inflammation. Intra-amniotic infection was defined as the concurrent presence of microbial invasion of the amniotic cavity and intra-amniotic inflammation. Sterile intra-amniotic inflammation was defined as the presence of intra-amniotic inflammation without microbial invasion of the amniotic cavity. Negative amniotic fluid was defined as amniotic fluid without intra-amniotic inflammation and microbial invasion of the amniotic cavity. The intra-amniotic inflammatory response was characterized by the concentration of IL-6 in the amniotic fluid. Maternal inflammatory response was determined by the concentrations of CRP concentrations and WBC counts in maternal blood. HCA was diagnosed based on the histological grades 3–4 for the chorion-decidua, and/or grades 3–4 for the chorionic plate, and/or grades 1–4 for the umbilical cord, and/or grades 1–4 for the amnion^44^. Funisitis was diagnosed based on histological grades 1–4 for the umbilical cord^44^. Inflammation of the amnion was diagnosed based on histological grades 1–4 for the amnion^44^. Compound neonatal morbidity was defined as the need for intubation, and/or respiratory distress syndrome, and/or transient tachypnea of newborns, and/or bronchopulmonary dysplasia, and/or retinopathy of prematurity, and/or intraventricular hemorrhage, and/or necrotizing enterocolitis, and/or intestinal perforation, and/or early-onset sepsis, and/or late-onset sepsis, and/or neonatal death before hospital discharge^20^.

### Statistical analyses

The demographic and clinical characteristics of the patients were compared using the non-parametric Kruskal–Wallis or Mann–Whitney *U* tests for continuous variables, as appropriate, and Fisher's exact or chi-square tests for categorical variables, as appropriate, and results were presented as median (interquartile range [IQR]) and number (%), respectively. The normality of the data was tested using the Anderson–Darling test. The loads of *Ureaplasma* spp. DNA in the amniotic fluid were not normally distributed; therefore, the non-parametric Mann–Whitney *U* test was used for the analyses. Kaplan–Meier survival curves were constructed, and a log-rank (Mantel-Cox) test was used to compare survival distributions among women with colonization, sterile intra-amniotic inflammation, and negative amniotic fluid. Spearman's partial correlation was used to adjust the results for gestational age at delivery. Differences were considered significant at *P* < 0.05. All *p*-values were determined using two-tailed tests, and all statistical analyses were performed using GraphPad Prism v8 for Mac OS X (GraphPad Software, San Diego, CA, USA) and the Statistical Package for the Social Sciences (SPSS), version 28.0.0.0, for Windows (SPSS Inc., Chicago, IL, USA).

## Supplementary Information

Supplementary Information.

## References

[1] Mercer BM (2003). Preterm premature rupture of the membranes. Obstet. Gynecol..

[2] Mercer BM (2005). Preterm premature rupture of the membranes: Current approaches to evaluation and management. Obstet. Gynecol. Clin. North Am..

[3] Waters TP, Mercer B (2011). Preterm PROM: Prediction, prevention, principles. Clin. Obstet. Gynecol..

[4] Cobo T (2012). Intra-amniotic inflammation predicts microbial invasion of the amniotic cavity but not spontaneous preterm delivery in preterm prelabor membrane rupture. Acta Obstet. Gynecol. Scand.

[5] Romero R, Ghidini A, Mazor M, Behnke E (1991). Microbial invasion of the amniotic cavity in premature rupture of membranes. Clin. Obstet. Gynecol..

[6] Romero R (1993). A comparative study of the diagnostic performance of amniotic fluid glucose, white blood cell count, interleukin-6, and gram stain in the detection of microbial invasion in patients with preterm premature rupture of membranes. Am. J. Obstet. Gynecol..

[7] Jacobsson B (2003). Microbial invasion and cytokine response in amniotic fluid in a Swedish population of women with preterm prelabor rupture of membranes. Acta Obstet. Gynecol. Scand..

[8] Cobo T (2012). Intra-amniotic inflammatory response in subgroups of women with preterm prelabor rupture of the membranes. PLoS ONE.

[9] DiGiulio DB (2010). Prevalence and diversity of microbes in the amniotic fluid, the fetal inflammatory response, and pregnancy outcome in women with preterm pre-labor rupture of membranes. Am. J. Reprod. Immunol..

[10] Kim YM (2004). Toll-like receptor-2 and -4 in the chorioamniotic membranes in spontaneous labor at term and in preterm parturition that are associated with chorioamnionitis. Am. J. Obstet. Gynecol..

[11] Kacerovsky M (2012). Soluble Toll-like receptor 1 family members in the amniotic fluid of women with preterm prelabor rupture of the membranes. J. Matern. Fetal. Neonatal. Med..

[12] Kacerovsky M (2012). Amniotic fluid soluble Toll-like receptor 4 in pregnancies complicated by preterm prelabor rupture of the membranes. J. Matern. Fetal. Neonatal. Med..

[13] Mantovani A, Garlanda C, Bottazzi B (2003). Pentraxin 3, a non-redundant soluble pattern recognition receptor involved in innate immunity. Vaccine.

[14] Galaz J (2020). Cellular immune responses in amniotic fluid of women with preterm prelabor rupture of membranes. J. Perinat. Med..

[15] Musilova I (2018). Amniotic fluid pentraxins: Potential early markers for identifying intra-amniotic inflammatory complications in preterm pre-labor rupture of membranes. Am. J. Reprod. Immunol..

[16] Romero R (2015). Sterile and microbial-associated intra-amniotic inflammation in preterm prelabor rupture of membranes. J. Matern. Fetal. Neonatal. Med..

[17] Jung E (2021). Bacteria in the amniotic fluid without inflammation: Early colonization vs. contamination. J. Perinat. Med..

[18] Romero R (2015). Sterile intra-amniotic inflammation in asymptomatic patients with a sonographic short cervix: Prevalence and clinical significance. J. Matern. Fetal. Neonatal. Med..

[19] Stranik J (2021). IgGFc-binding protein in pregnancies complicated by spontaneous preterm delivery: A retrospective cohort study. Sci. Rep..

[20] Kacerovsky M (2020). Lactobacilli-dominated cervical microbiota in women with preterm prelabor rupture of membranes. Pediatr. Res..

[21] Musilova I (2015). Intraamniotic inflammation in women with preterm prelabor rupture of membranes. PLoS ONE.

[22] Menon R, Behnia F, Polettini J, Richardson LS (2020). Novel pathways of inflammation in human fetal membranes associated with preterm birth and preterm pre-labor rupture of the membranes. Semin. Immunopathol..

[23] Musilova I (2017). Maternal white blood cell count cannot identify the presence of microbial invasion of the amniotic cavity or intra-amniotic inflammation in women with preterm prelabor rupture of membranes. PLoS ONE.

[24] Larsen B, Hwang J (2010). Mycoplasma, ureaplasma, and adverse pregnancy outcomes: a fresh look. Infect. Dis. Obstet. Gynecol..

[25] Donders GGG, Ruban K, Bellen G, Petricevic L (2017). Mycoplasma/ureaplasma infection in pregnancy: To screen or not to screen. J. Perinat. Med..

[26] Sprong KE, Mabenge M, Wright CA, Govender S (2020). Ureaplasma species and preterm birth: Current perspectives. Crit. Rev. Microbiol..

[27] Jacobsson B, Aaltonen R, Rantakokko-Jalava K, Morken NH, Alanen A (2009). Quantification of ureaplasma urealyticum DNA in the amniotic fluid from patients in PTL and pPROM and its relation to inflammatory cytokine levels. Acta Obstet. Gynecol. Scand..

[28] Kacerovsky M (2014). Bedside assessment of amniotic fluid interleukin-6 in preterm prelabor rupture of membranes. Am. J. Obstet. Gynecol..

[29] Kacerovsky M (2012). Intraamniotic inflammatory response to bacteria: Analysis of multiple amniotic fluid proteins in women with preterm prelabor rupture of membranes. J. Matern. Fetal. Neonatal. Med..

[30] Romero R (2015). Evidence of perturbations of the cytokine network in preterm labor. Am. J. Obstet. Gynecol..

[31] Peiris HN (2020). Prostaglandin and prostamide concentrations in amniotic fluid of women with spontaneous labor at term with and without clinical chorioamnionitis. Prostaglandins Leukot Essent Fatty Acids.

[32] Kim CJ (2015). Acute chorioamnionitis and funisitis: definition, pathologic features, and clinical significance. Am. J. Obstet. Gynecol..

[33] Rodriguez-Trujillo A (2016). Gestational age is more important for short-term neonatal outcome than microbial invasion of the amniotic cavity or intra-amniotic inflammation in preterm prelabor rupture of membranes. Acta Obstet. Gynecol. Scand.

[34] Kacerovsky M (2020). Amniotic fluid glucose level in PPROM pregnancies: a glance at the old friend. J. Matern. Fetal Neonatal. Med..

[35] Kacerovsky M (2021). Cervical Gardnerella vaginalis in women with preterm prelabor rupture of membranes. PLoS ONE.

[36] Kacerovsky M (2020). Antibiotic administration reduces the rate of intraamniotic inflammation in preterm prelabor rupture of the membranes. Am. J. Obstet. Gynecol..

[37] Matulova J (2021). Birth weight and intra-amniotic inflammatory and infection-related complications in pregnancies with preterm prelabor rupture of membranes: A retrospective cohort study. J. Matern. Fetal. Neonatal. Med..

[38] Fraunberger P (1998). Validation of an automated enzyme immunoassay for Interleukin-6 for routine clinical use. Clin. Chem. Lab Med..

[39] Stranik, J. *et al.* Intra-amniotic infection and sterile intra-amniotic inflammation are associated with elevated concentrations of cervical fluid interleukin-6 in women with spontaneous preterm labor with intact membranes. J. Matern. Fetal. Neonatal. Med. 10.1080/14767058.2020.1869932 (2021). 10.1080/14767058.2020.1869932 33412979

[40] Musilova I (2016). Ureaplasma species and mycoplasma hominis in cervical fluid of pregnancies complicated by preterm prelabor rupture of membranes. J. Matern. Fetal. Neonatal. Med..

[41] Fouhy F (2015). The effects of freezing on faecal microbiota as determined using MiSeq sequencing and culture-based investigations. PLoS ONE.

[42] Musilova I (2017). Maternal serum C-reactive protein concentration and intra-amniotic inflammation in women with preterm prelabor rupture of membranes. PLoS ONE.

[43] Stepan M (2016). Maternal serum C-reactive protein in women with preterm prelabor rupture of membranes. PLoS ONE.

[44] Salafia CM, Weigl C, Silberman L (1989). The prevalence and distribution of acute placental inflammation in uncomplicated term pregnancies. Obstet. Gynecol..

[45] Chalupska M (2021). Intra-amniotic infection and sterile intra-amniotic inflammation in cervical insufficiency with prolapsed fetal membranes: clinical implications. Fetal. Diagn. Ther..

[46] Musilova I (2020). Interleukin-6 measured using the automated electrochemiluminescence immunoassay method for the identification of intra-amniotic inflammation in preterm prelabor rupture of membranes. J. Matern. Fetal. Neonatal. Med..

